# Does Inflammation Determine Whether Obesity Is Metabolically Healthy or Unhealthy? The Aging Perspective

**DOI:** 10.1155/2012/456456

**Published:** 2012-10-04

**Authors:** Iftikhar Alam, Tze Pin Ng, Anis Larbi

**Affiliations:** ^1^Tubingen Aging and Tumor Immunology (TATI) Group, University of Tubingen (ZMF), Waldhornlestrasse 22, 72072 Tubingen, Germany; ^2^Faculty of Agriculture, Abdul Wali Khan University Mardan, Khyber Pakhtunkhwa (KPK), Mardan 25000, Pakistan; ^3^Department of Psychological Medicine, National University Health System, Gerontological Research Programme, National University of Singapore, Singapore 119228; ^4^Singapore Immunology Network (SIgN), Biopolis, Agency for Science, Technology and Research (A∗STAR), Singapore 138648

## Abstract

Obesity is a major health issue in developed as well as developing countries. While obesity is associated with relatively good health status in some individuals, it may become a health issue for others. Obesity in the context of inflammation has been studied extensively. However, whether obesity in its various forms has the same adverse effects is a matter of debate and requires further research. During its natural history, metabolically healthy obesity (MHO) converts into metabolically unhealthy obesity (MUHO). What causes this transition to occur and what is the role of obesity-related mediators of inflammation during this transition is discussed in this paper.

## 1. Background

**Figure 1:**
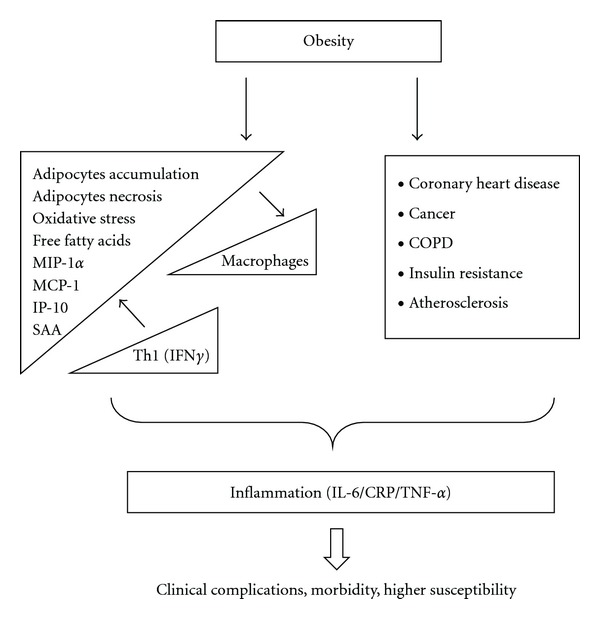
The relationship between obesity and comorbidities. The consequences of obesity are depicted and include the immunological component (T cells and macrophages) that drives inflammation together with adipocytes. The associated pathologies mentioned have been associated with increased levels of inflammatory markers that are also increased in obesity. Although it is not clear which one is the consequence and which one is the cause both are associated to higher clinical vulnerability. MIP-1*α*: macrophage inflammatory protein-1 alpha; MCP-1: monocyte chemotactic protein-1; IP-10: interferon gamma-induced protein 10; SAA: serum amyloid A; Th1: T helper 1 cytokine; COPD: chronic obstructive pulmonary disease.

Obesity and its associated comorbidities have become major health problems in the world [1]. The association between obesity and the development of major complications in acute pancreatitis [2], fatty liver diseases [3], vascular inflammation and coronary heart disease [4, 5], chronic obstructive pulmonary disease [6], risk of cerebral ischemia and brain injury [7], atherosclerotic vascular disease and myocardial infarction [8], and cancers [9–11] are strongly linked to chronic inflammation (Figure 1). In particular, insulin resistance, a direct or indirect result of obesity, is characterized by a chronic state of subclinical inflammation [12] and inactivation of a number of inflammatory mediators [13–15]. Elevated serum concentrations of C-reactive protein [16], interleukin IL-6, IL-8, and tumor necrosis factor (TNF)-*α* are observed in obese individuals with elevated insulin resistance [17]. 

Interestingly, however, a life course perspective on obesity recognizes that obese individuals are not a homogeneous group, but highly heterogeneous, given individual differences in terms of health status and functional ability among obese individuals. A substantially large number of the adult obese population is reported to remain relatively “metabolically healthy” obese (MHO) [18]. Unlike “metabolically unhealthy” obese (MUHO) individuals, MHO individuals demonstrate an absence of impaired glucose tolerance, dyslipidemia, hyperuricemia, and hypertension [19]. Whether these represent valid subtypes of obesity, and whether obesity is an independent predictor of metabolic outcomes or mediated by inflammatory status is still unclear. Research so far has not elucidated the factor(s) responsible for causing obesity to become metabolically unhealthy. These are important questions since a lifetime burden of obesity and its consequences, and a clear understanding of the metabolic transition process carries a host of clinical and public health implications.

## 2. The Disease Burden of Obesity

Obesity is a condition of energy imbalance between intake and expenditure. When an organism encounters food excess, it conserves the nutrients either as glycogen for short-term storage or as lipids for longer storage duration, which may result in a consequent state of obesity. The mechanisms underlying these biological changes are beyond the scope of this paper and the readers are directed to some excellent work elsewhere [20, 21].

Human obesity is one of the oldest reported health disorders and can be traced as early as 25,000 years; nevertheless, it still remains relatively a rare condition for most of the human history [22]. However, due to unparalleled socio-demographic trends over the recent past, there has been a drastic increase in the prevalence of obesity in most regions of the world and particularly in developed countries [23–25]. It was previously estimated that at least 1.1 billion people across the globe are overweight and 312 million of them are obese [26]. World Health Organization (WHO) has estimated that by the end of 2030, there will be approximately 370 million people suffering from obesity and its associated comorbidities [27]. In fact, the prevalence of adult obesity, as defined by a body mass index (BMI) in excess of 30 kg/m^2^, has been escalated to such a degree that a normal BMI (18.5–24.9 kg/m^2^) is no longer the norm, as only a small minority of the population will fall into this category. It is now apparent that most countries of the world, even those which have just recently struggled with undernutrition (underweight), and some that continue to do so [28], are experiencing increases in obesity prevalence. The face of the fact is that the epidemic of obesity has now spread throughout most of the civilized world rendering it more appropriate to term it the “obesity pandemic.” Although a number of potential factors have been suggested to explain the obesity pandemic, including increased sedentary lifestyle—particularly a decrease in occupational physical activity [29], increased dietary caloric intake and alterations in diet composition [24], smoking [30], and others [31], the exact causes of the obesity pandemic are still in doubts and continue to be debated.

## 3. Obesity-Induced Inflammation

Obesity in humans is associated with low-level inflammation [32–37]. In this respect, obesity may be viewed as a form of chronic inflammation [38]. The inflammatory response triggered by obesity involves a number of well-known components of the typical classical inflammatory response to pathogens. These components include (i) systemic increases in circulating inflammatory cytokines and adipokines and acute phase proteins, (ii) recruitment of leukocytes to inflamed tissues, (iii) activation of tissue leukocytes, and (iv) generation of reparative tissue responses (Figure 1). However, the nature of obesity-induced inflammation is unique in comparison to other inflammatory paradigms of infections, autoimmune diseases, and others of the type. Qualitatively, for example, in chronic obesity, a low-grade activation of the innate immune system is produced that affects steady-state measures of metabolic homeostasis over time. Hence obesity-associated inflammation is characterized by a low-level but chronic inflammatory state [39]. On the other hand, in response to pathogens or acute injury, inflammation seems to be robust but resolves very quickly once the threat is removed. 

A great number of metabolic disorders are caused by obesity. Among them, insulin resistance (Figure 1) has been shown to be the most important, associated with a chronic state of subclinical inflammation, and characterized by increased serum concentrations of C-reactive protein [16]. In addition, interleukin (IL)-6, IL-8, monocyte chemotactic protein (MCP)-1, and tumor necrosis factor (TNF-*α*) have also been found to be increased with elevated insulin resistance [17, 40, 41] along with factors such as serum amyloid A, resistin, leptin, and adiponectin [42, 43]. These may result in a large number of associated complications that have been studied and reviewed extensively [2–11]. 

There are 24 different adipokines, which have been reported in relation to body fat and obesity [44]. The levels of these adipokines are usually elevated in obese humans, and circulating concentrations of each of these factors increase with the degree of obesity. Except for IL-1, the majority are secreted by adipocytes themselves [43, 45, 46]. Serum amyloid-A is an adipokine secreted by adipocytes, that can act directly on macrophages to increase its production of inflammatory cytokines such as TNF-*α*, IL-1, and IL-6, and resistin [43, 47]. Most of these adipokines are inflammatory proteins (such as IL-8, PAI-1, MCP-1, IL-6, IL-1Ra, TNF*α*, sTNF RII, and IL-18), while a few adipokines such as CRP, haptoglobin, and amyloid A are actually acute phase proteins primarily released by the liver in response to mild inflammatory response associated with human obesity. How and why these adipokines are elevated in serum in obesity is, however, still very much unclear. Furthermore, obesity is also associated with macrophage accumulation in adipose tissue [48], and there is evidence that certain factors secreted by adipocytes can act in an endocrine-like manner to activate monocytes as well (Figure 1). For example, resistin can induce cytokine production in macrophages, and its production is also regulated by the inflammatory cytokines TNF-*α*, IL-1, and IL-6, creating a potential positive feedback loop [49, 50]. Leptin has the capability to exert an endocrine effect to increase cytokine production in blood monocytes [51]. Therefore, a continuous cycle of cross-talk between adipocytes and monocytes [52] may stimulate and perpetuate the proinflammatory status associated with obesity. 

## 4. Inflammatory Signaling and Mediators of Inflammation in Obesity

Once it was discovered that there is an increased expression of the proinflammatory cytokine (TNF*α*) in adipose tissue in obese mice, much research was focused and turned on the key roles of inflammatory mediators in obesity [40]. It is now well established that changes in inflammatory signaling by adipocytes and infiltration of adipose tissue by immune cells are the key features of obesity-induced insulin resistance and a number of associated metabolic diseases [16, 48, 53]. 

It has been shown in obese mice that both adipocytes and macrophages residing in adipose tissue secrete a number of cytokines including TNF*α*, IL-6, IL-1, and migration inhibitory factors [53]. Increased expressions of inflammatory mediators (primarily cytokines but also adipokines) have also been observed in visceral fat of obese human individuals [53, 54]. Some of these cytokines have been shown to cause a disruption in insulin signaling. This disruption is attributed to a multitude of mechanisms, including induction of the suppressors of cytokine signaling family of proteins, which have been shown to inhibit insulin receptor kinase activity [55, 56]. Several other *in vitro* and *in vivo* studies suggest that TNF-*α*, IL-1, and IL-6 can each directly impair insulin sensitivity by interfering with insulin-stimulated glucose uptake in peripheral tissues [57–59].

Cytokines have also been reported to activate inflammatory signaling via c-Jun N-terminal kinase (JNK) and inhibitor of kappa kinase (IKK) pathways in both immune and neighboring nonimmune cells [60, 61]. This results in increased inflammation and direct inhibition of insulin action, as well as possible alterations in other metabolic targets, that in combination contribute to overall metabolic deterioration. The negative impact of inflammatory pathway activation on carbohydrate metabolism has been studied in mice [62]. Although the nature of detailed mechanisms underlying these pathways is still in doubts, there have been some reports of the possible causes of this impact on carbohydrate metabolism that are attributed to deletion of either JNK [61] or inhibition of IKK [13, 60, 63] or even neutralization of TNF-*α* [40] or IL-1 [64]. These mechanisms collectively may lead to a situation of decreased inflammatory signaling and improved insulin responsiveness and glucose tolerance. Immune and neighboring nonimmune cells have been shown to be important in the observed improvements in glucose homeostasis [13, 15, 60, 65]. Of particular note, manipulation of levels of these inflammatory mediators can impact insulin resistance and other metabolic parameters whether it is adipose cell accumulation in muscle or lipid accumulation in liver [16]. This may be an indication that alterations in immune signaling are triggered by excess adiposity, which may be essential mediators of the metabolic dysfunction observed in obesity. 

Macrophage infiltration has also been observed in skeletal muscle, suggesting that local inflammatory signaling could also directly influence muscle insulin resistance [66]. It is important to note that in many experimental systems, muscle effects appear to emerge secondary to alterations in other organs, including adipose tissue and liver [67]. However, muscle-specific expression of the anti-inflammatory cytokine IL-10 has been shown to greatly improve muscle insulin sensitivity, consequently yielding reduced inflammation in this tissue despite normal development of obesity when fed a high-fat diet [68]. In addition, increased inflammatory signaling in the brain has also been observed in response to overnutrition, or in the context of obesity, resulting in improper regulation of energy uptake and energy expenditure by peripheral tissues [69]. Thus, it appears that increased inflammation is a systemic feature associated with surplus energy intake. Therefore interventional or therapeutic solutions for reducing inflammatory signaling induced by metabolic stress would, therefore, be necessarily expected to improve on systemic energy homeostasis at multiple tissue and organ or system levels.

## 5. Metabolically Healthy and Unhealthy Phenotypes of Obesity

### 5.1. Metabolically Healthy Obesity

**Figure 2:**
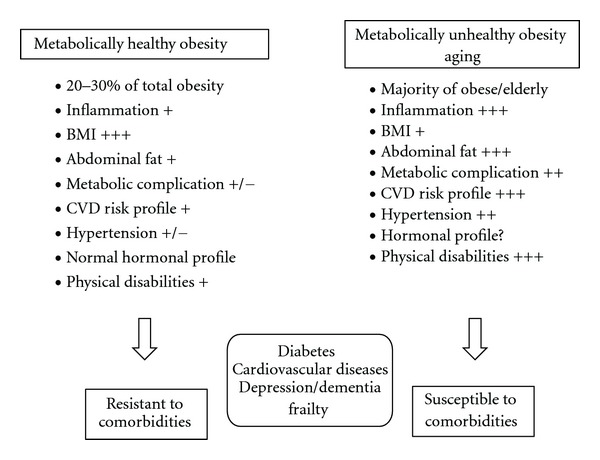
Differences between metabolically healthy and unhealthy obesity and similarities with aging. The hallmarks of metabolically healthy obesity are depicted and associate poorly with comorbidities while metabolically unhealthy obesity shares similarities with the aging process and are both associated with increased prevalence to pathologies and chronic conditions. BMI: body mass index; CVD: cardiovascular diseases.

Not all obese individuals exhibit increased risk of inflammation and not all normal-weight individuals are metabolically healthy or free from CVD [70]. Two distinct subtypes of obesity have been proposed, referred to in different ways by various authors. One type is “metabolically healthy” and the other is “metabolically unhealthy” obese (MHO and MUHO, resp.). It is interesting to note that approximately 20–30% of the adult obese population remains at the level of relatively “metabolically healthy” obesity (MHO) [18, 19, 70] as compared to those with “metabolically unhealthy” obesity (Figure 2). Individuals in the first subtype have also been termed “metabolically normal obese” [71], “metabolically healthy but obese” [70], “obese metabolically normal” [72] or described as having metabolically benign obesity [73] or uncomplicated obesity [74]. MHO individuals exhibit increased levels of body mass index (BMI) and body fat (BF) but no other metabolic complications [72]. Significantly, both subtypes associate with different inflammatory profiles. MUHO exhibit increased levels of inflammation compared to other normal-weight individuals [75], while MHO exhibit reduced levels of inflammation compared to other obese individuals [76]. MHO individuals may display an absence of impaired glucose tolerance, dyslipidemia, hyperuricemia, and hypertension [19]. In addition, their metabolic and CVD risk profiles are relatively mild [77], with high levels of insulin sensitivity [71], absence of hypertension [78], normal lipid, inflammation, and hormonal profiles [73], and importantly a favorable immune profile [79]. It has been suggested that the unique metabolically healthy subgroup of obese individuals appear to be protected or more resistant to the development of comorbidities associated with obesity [70–72, 80].

Despite the knowledge of the MHO phenotype for substantially longer time now, there still currently exist no established criteria for the definition of MHO individuals [80]. Some have used arbitrary cut-points of insulin sensitivity [19], cardiometabolic risk factor clustering [73], or the complete absence of any metabolic aberration [74] to delineate MHO from MUHO individuals. Furthermore, no convincing answers on how the mechanism is in place which provides protection against a number of diseases in MHO individuals. Several hypotheses have been proposed in an attempt to explain the role of the adipose tissue in the metabolic dysfunction associated with obesity in order to better understand the difference between the two distinct groups (Figure 2). Thus, the altered pattern of adipokine secretion by the obese adipose tissue [81, 82], the inflammatory state associated with obesity [83], or the inability of the adipose tissue to expand its mass in response to increased energy intake [84] have all been signaled as possible culprits. However, all these different hypotheses are not mutually exclusive. As a plausible mechanism for the normal cardiometabolic profile, it has been reported that for the same BMI, MHO subjects tend to have a lower waist circumference [19], and specifically less VAT accumulation [71] in contrast to MUHO individuals, therefore, closely resembling the metabolically benign gynoid obesity phenotype. Some recent studies have shown that increased visceral/abdominal fat (characteristics of MUHO) is positively associated with metabolic disease [85, 86], independent of overall adiposity [87, 88]. Similarly, high thigh intermuscular fat is associated with poorer glucose tolerance. On the other hand, subcutaneous thigh fat (characteristic of MHO) is associated with more favorable levels of glucose and lipids [89, 90]. Another explanation for the more favorable metabolic profile of some obese people may be related to inflammatory status [91]. 

Another critical unanswered question is why and how MHO individuals may differ from MUHO regarding the inflammatory mediators in obesity. Inflammatory markers, such as IL-6, TNF-*α* and other cytokines and adipokines such as resistin and adiponectin are associated with metabolic alterations [92, 93]. These adipocytokines are closely linked to abdominal obesity, particularly to visceral adipose tissues, while some evidence suggests that thigh subcutaneous fat is related to more favorable inflammatory profiles [94–96]. Overall, the unique protective mechanism in these individuals is attributable in part to a reduced inflammatory profile [77], and uncoupling of inflammatory signal transduction from obesity-driven inflammatory response [97, 98].

Theoretically, MHO and MUHO may represent distinct subtypes of obesity that were predetermined genetically to confer differing metabolic and cardiovascular risks. Another theoretical possibility is that MHO and MUHO represent transitions phases from nonobesity in the development and natural history of obesity, with MHO individual ultimately turning into MUHO. It is tempting to think over the possibility of the other way round, that is, the possibility of converting MUHO into MHO. Whether MHO is sustained or not for substantially a longer period may depend upon a number of factors, including the levels of cytokines and/or adipokines. A major challenge is to understand both the initiating signals and downstream mechanisms involved in the establishment of inflammation that occurs during these transitions from relatively healthy form of obesity into unhealthy one. Further studies are needed to investigate the differences in concentrations of these inflammatory factors and to establish whether it is an imbalance in the concentrations of these cytokines that causes this transition or whether the transition to MUHO occurs first due to various other factors and then brings about changes in the levels of these cytokines (which came first, the chicken or the egg?). The answer to this question may have paramount clinical and public health implications. Once the temporal relationship is established, the course of obesity can be stopped or delayed at some stage during its natural history before it becomes “metabolically unhealthy.” 

One possible way to investigate the mode of this transition is to study obesity over enough time to observe the impact of its related chronic inflammation. Thus, after the 5th decade may be the best time to study such transitions, for a number of reasons. First, this is the age where an individual starts entering into old age, particularly in the developing countries [99]. Second, most of the changes in hormonal output and functions [100], life-style behaviors [101], diet and nutrition, body composition [99, 102, 103], and immunological alterations [104, 105], and overall physical, physiological, and immunological parameters [106, 107] are likely to emerge significantly differently in the elderly with an overall impact on obesity and the inflammatory mediators. Third, obesity is more prevalent in older age. Finally, part of the comorbidity associated with obesity is common to aging (Figure 2) and may suggest similar mechanisms. In relation to obesity, the transition between MHO and MUHO is likely to occur from adulthood-to-early old age and hence it may be wise to focus on obesity and its related issues at this age. 

In the last sections of this paper, an overview of metabolically healthy and unhealthy obesity in the context of aging is presented followed by a description of obesity- and age-associated inflammation.

### 5.2. Metabolically Healthy Obesity in Old Age

A clear distinction between MHO and MUHO is even of great clinical implication in the aged subjects as ageing populations face the challenges of both rising numbers of the elderly and increasing obesity prevalence. In addition, elderly subjects may present with differences in the etiology, pathogenesis and prevalence of obesity and its comorbidities and related inflammatory patterns and implications.

It is important to note that the prevalence of overweight and obesity is increasing among older age groups in developed countries [108]. The prevalence of obesity increases from 15 to 20% at puberty to 40% in 60–69 years old individuals [109, 110], which is also a rational for studying obesity in this age group in particular. The problem of obesity in the elderly thus has great relevance and requires more research and clinical attention. 

Research studies so far have not considered in greater depth the phenotypic heterogeneity of obesity such as the metabolically healthy and unhealthy subtypes, particularly in the aged populations. In virtually all studies, measures of adiposity were investigated alongside metabolic risk factors and individual components of the metabolic syndrome as independent risk factors predicting health outcomes. The health risks of overweight-obese individuals without metabolic risk factors and those with metabolic risk factors have been investigated, to the best of our knowledge, in one study so far [111]. This longitudinal study assessed risk for diabetes or cardiovascular disease (CVD) stratified by body mass index (BMI) and the presence or absence of metabolic syndrome (MetS) or insulin resistance (IR). It found that metabolic risk factor clustering or IR appeared to confer much of the risk for diabetes or CVD commonly associated with elevated BMI. Of note, among people older than 70 years, there is decline in obesity prevalence probably due to selective mortality of people in middle ages, such that relatively fewer obese people survive into older ages. Aging is also associated with emergence of frailty and sarcopenia which are partly defined by weight loss, especially fat-free muscle. Thus, very old individuals may convert from obesity defined by increased BMI to obesity due to reduced muscle/fat ratio but with a normal BMI. In connection to that, the normal-weight obese (NWO) syndrome in the elderly subjects of normal body weight and BMI has been identified (Figure 2). Interestingly, the fat mass in these subjects was 30% of their total body weight and these subjects had a likelihood of increased risk of developing obesity-related diseases [112]. The data suggests that in the elderly obesity should be redefined as the amount of excess fat storage associated with elevated health risk. The relationship between overweight/obesity and total mortality in the elderly is controversial [113]. The expected increase on mortality associated with increasing BMI is not observed in many older population studies, and a number of studies, in fact, observed a U-shaped curve describing the relationship between BMI and mortality in old age [114]. Higher BMI values were associated with a smaller relative mortality risk in elderly persons compared with young and middle-aged populations [115]. These data suggest that use of BMI alone as the sole indices of excess adiposity in studies of older populations is a contributing factor for discrepant findings. On the other hand, studies using indices of amount and distribution of fat tend to consistently show a greater association with specific and total mortality in the elderly than BMI alone. Larger waist circumference or waist-to-hip ratio (relatively stronger indicators for adiposity) has been shown significantly associated with mortality in older subjects, whilst BMI alone was not. Thus, central adiposity and relative loss of fat-free mass may be more important than BMI in determining the health risk associated with obesity in older ages. The interpretation of the relationship between adiposity and mortality in the elderly is complicated by the difficulty in accounting for selection and confounding factors (such as survival bias, smoking, and physical inactivity), but is probably also inherently complex. 

Numerous studies have shown that overweight/obesity is associated with a host of known nonfatal health outcomes, mostly in the aged populations [116–122]. Hypothetically, MUHO might be responsible for much of the association with metabolic syndrome, diabetes, cardiovascular disease, and other obesity-related metabolic complications including depression and dementia; on the other hand, MHO might still be associated with similar risk of other related complications such as physical limitations and disability, osteoarthritis, and obstructive sleep apnea. As with total mortality, a J-shape relation between BMI and disability in older persons has been reported [118] with observed greater disability for both low and high values of BMI in both sexes. The relationships with various morbidity outcomes are particularly stronger with indices of central adiposity than BMI, and some indeed showed associations independently of BMI. Taking another case in point, although some studies showed a significant negative relationship between high BMI and depressive symptoms (Jolly Fat hypothesis) [123, 124] other studies showed that increased waist circumference was either not associated or positively associated with depressive symptoms [125, 126].

## 6. Aging and Inflammation

In the previous sections, obesity and its different phenotypic forms were reviewed with emphasis of possible differences due to natural aging. The consequences related to MHO and MUHO are likely to be greatly affected by aging as a variety of disorders and infections are unique to the elderly, which may additionally exacerbate the effects of obesity. Thus, aging seems to present with a great variety of patterns and unique sets of obesity and age-related infections and diseases. It is, therefore, very tempting and urging to study obesity, inflammation, and the related comorbidities in the context of aging. 

**Figure 3:**
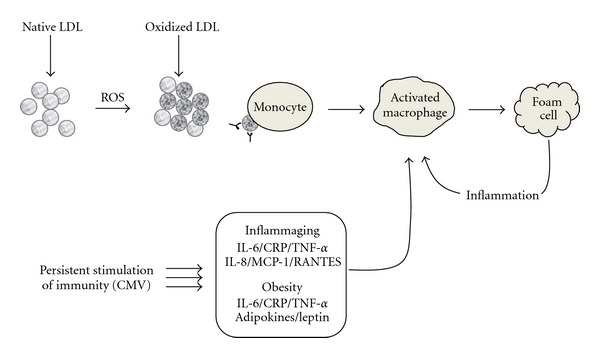
Conversion of naïve LDL to oxidized LDL and formation of foam cells from monocytes: implication of aging and obesity in sustaining/worsening inflammation. A typical example of how chronic condition can participate in the development of diseases. In this case, atherosclerotic plaque formation is increased by parallel proinflammatory signals derived from obesity and/or inflammaging. LDL: low-density lipoprotein; ROS: reactive oxygen species; CMV: cytomegalovirus; RANTES: regulated and normal T cell expressed and secreted.

In general, inflammation is a very tightly regulated process that ensures recruitment of competent and experienced cells, clearance of pathogen with minimal tissue damage and sequels. The duration, intensity and variety of its components will determine the impact of inflammation on health including the cardiovascular, bone/joint, respiratory and immune systems. A lack of balance in this equilibrium may lead to significant consequences locally (tissue site) or in a systemic manner. Inflammation is the most necessary step for recovery and most cells produce cytokines/chemokines/adipokines and soluble mediators of inflammation. Not only immune cells but also endothelial cells, fibroblasts, and keratinocytes among other cell types are able to produce these mediators and are partners and players of the immune responses, fully involved in the inflammatory process. One example is the atherosclerotic plaques that develop over years. There is no doubt in the scientific community regarding the involvement of monocytes/macrophages in the formation of these plaques. The increase in oxidized low-density-lipoproteins (oxLDL) and its associated secretion of proinflammatory molecules from the endothelium is responsible for the initial recruitment of immune cells (Figure 3). The molecular events leading to LDL oxidation and subsequent effects are extensively reviewed by Leonarduzzi et al. [127]. Briefly, the monocytes differentiate into macrophages, which possess a high propensity to uptake oxLDL. The uncontrolled accumulation of the oxLDL initiates the formation of lipid droplets within the monocytes/macrophages, ultimately leading to the formation of foam cells. These foam cells in turn release proinflammatory mediators that sustain this process. The accumulation of foam cells leads to the formation of a physical barrier in the vessels characterizing atherosclerosis. This pathology is a process that requires years or decades to be observed clinically. For this reason, many consequences of uncontrolled inflammation are seen later in life, that is, in the elderly population. Recent animal models (zebra fish) allow to now study and modulate the effect of oxLDL in a shorter period of time [128], which should enable to improve our understanding of relationship between inflammation and obesity-related diseases.

Cardiovascular diseases (CVD), including heart and vascular disease and atherosclerosis, are highly prevalent, particularly in the old age. Inflammation induced by obesity accelerates atherosclerosis. Adipose tissue and adipocytes produce leptin and large numbers of a variety of other hormones, peptides, and other molecules that affect cardiovascular function. The production of these substances is supposed to be through distinct endocrine, autocrine and paracrine mechanisms and believed to lead to cytokine-mediated inflammatory changes in the liver and systemic inflammation and atherosclerosis [129]. CVD and the related heart diseases are recognized as chronic inflammatory conditions of the blood vessels that result from the excessive transendothelial passage (transcytosis) of cholesterol rich atherogenic lipoproteins (VLDL, IDL, and LDL) from the plasma into the intima. Once there, these lipoproteins are likely to be retained in the subendothelial spaces leading to infiltration of macrophages and T cells into these spaces and ultimately interact not only with each other but also with the cells of the arterial wall [130–132], forming foam cells (FC) in the vicinity [133], which is considered as the first step of plaque formation (Figure 3). FC are generated from altered and/or oxidized LDL, termed as modified LDL. The rate and intensity of FC formation depends upon the relative size and shape of LDL [134] as LDL comprises a group of very heterogeneous particles, which comprise multiple distinct subclasses that differ in size, density, physicochemical composition, metabolic and oxidative behavior, as well as atherogenicity [135]. Increasing evidence suggests that size and density of LDL have a direct influence on cardiovascular risk [136]. There is also evidence suggestive of the coexistence of proatherogenic LDL subclasses and elevated inflammation [137]. For instance, elevated levels of IL-18 are associated with reduced LDL size, independently of other inflammatory and metabolic risk factors [138]. There is a close relationship between atherogenic small, dense LDL and inflammation [139] and a reduction in the concentrations of these LDL has been shown to lead to a strong reduction in CVD-associated morbidity and mortality rate [140–142].

Based on the observations of greater than expected reduction in CVD events in the JUPITER study (Justification for the Use of Statins in Primary Prevention: An Intervention Trial Evaluating Rosuvastatin) [143], it has been suggested that other factors besides LDL-cholesterol may also be involved in the reduction of CVD event. Therefore the modulation of these factors, particularly of inflammation and atherogenic lipoproteins, represents a main target of CVD prevention [144]. In addition, there is overwhelming evidence supporting the pathogenetic role of fibrinogen and CRP in atherosclerosis [145] and their predictive capability for future cerebro- and cardiovascular events in patients with subclinical and clinical atherosclerosis [146–148].

Obesity and type 2 diabetes (T2D) are probably inter-twined. Unfortunately, however, no direct study has ever provided the evidence that obesity can cause diabetes. Interestingly, reports published by Solinas et al. [15] indicate that inflammation could be the key factor causing obesity-induced type 2 diabetes. The group has indicated that obesity without inflammation is not prominently associated with insulin resistance and/or diabetes. In addition, gestational diabetes (GDM) is very common and high prevalence rates have been reported from both developed and developing countries [149–152]. Women with previous gestational diabetes (pGDM) are characterized by chronic subclinical inflammation which is associated with insulin resistance and abnormality in glucose metabolism. Approximately 30% of these women have metabolic syndrome and many of them will develop T2DM within 5 years of diagnosis. The conversion rates from GDM to T2DM range from 2.6% to 70% over a period of 6 weeks to 28 years postpartum [153].

As described in the previous section, obesity is not restricted to younger populations. However, the intensity of obesity-related inflammation may not be the same over age. Also, obesity-related or unrelated inflammation may not be similarly handled in young versus elderly individuals. This raises the importance of understanding the unbalance in inflammation during aging. Two types of unwanted inflammation must be considered in aging (i) acute inflammation resulting from bad management of the inflammatory process, which in turn results from immune responses, and (ii) chronic inflammation resulting from persistent condition(s) in the elderly including age-specific infections such as cytomegalovirus CMV. This later is often referred to as low-grade inflammation or even “inflammaging” as coined by Franceschi et al. [154], who identified a variety of these proinflammatory mediators in aging [155], namely, RANTES, MIP-1*α*, IL-8, MCP-1, IL-6, and TNF-*α* amongst others. Individually these mediators are involved in the development of many subclinical and clinically assessable events such as atherosclerosis, dementia, and diabetes (Figure 3). Not only is the presence of these markers in higher quantity linked to comorbidities but also to mortality [155]. The unbalance between IL-6/TNF-*α* levels and IGF-I levels in the elderly directly accounts for the loss of fat-free mass suggesting that obesity and sarcopenia are highly inflammation dependent [156]. An important set of markers is IL-6, TNF-*α*, and CRP. This triad is involved in much comorbidity and has shown significant correlations with mortality [157]. The increased levels of such inflammatory mediators are not observed in all elderly individuals. Many mechanisms influence how inflammation is resolved and many events will induce such inflammation. Genetics lifestyle and immunological history are probably the most important players in the inter-individual differences [158]. Immunological history refers to the collection of events that lead to an immune response. This response may result in clearance of infection (acute response) or may simply resolve primary infection without clearing the pathogen. This later, chronic/persistent infection is a major issue for the immune system as it requires life-long immunosurveillance and dedicated resources. Pathogens requiring life-long control can be viruses (HIV, Cytomegalovirus), bacteria (*H. pylori*, M. tuberculosis), or parasites (P. falciparum, T. gondii). HIV has been shown to deeply impact on body composition such as increased visceral adipose tissue even with therapies. Such changes were also associated to increased susceptibility to atherosclerosis and associated complications suggesting that inflammation may be a corner stone in these processes [159]. 

*Helicobacter pylori* is now easily eradicated following its identification, however, there is evidence showing that even after resolution of infection there is no reduction in the incidence of gastric cancers suggesting that the inflammation initiated has long-lasting effects suggesting that immunological history in this case have irreversible effects [160]. While the infection with HIV, parasites, and even *H. pylori* may be reduced greatly in the next decades and already low in developed countries, a majority of individuals encounter CMV during their life. The seroconversion ranges between 0.5 and 1.5% per year [161] but may be very high in younger populations, especially in developing countries. Thus, such a persistent infection may impact on individuals' health for decades. One of the most important studies on the relationship between CMV seropositivity and mortality was performed recently in a US-based platform [162]. Individuals aged 25 and above from the NHANES III (started in 1988) were followed-up for mortality and adjustment for multiple confounders revealed that CMV seropositivity was statistically significantly associated with all-cause mortality. Individuals with high CRP levels showed a 30% higher risk for all-cause and cardiovascular diseases-mortality compared to those with low CRP levels. That study however did not investigate the putative synergy of obesity and CMV seropositivity on all-cause mortality.

## 7. Conclusion

Obesity in humans is a unique form of low-grade inflammation. In chronic obesity, a low-grade activation of the innate immune system is provoked affecting metabolic homeostasis steadily. In contrast to the inflammatory response to pathogens or acute injury which is robust but resolves very quickly once the threat is removed, inflammation in obesity seems to sustain for relatively longer period of time. Inflammatory state of obesity is characterized by insulin resistance, increased serum concentrations of C-reactive protein, IL-6, IL-8, TNF-*α*, and so forth. Obesity-induced insulin resistance also causes changes in inflammatory signaling by adipocytes and infiltration of adipose tissue by immune cells. Alterations in immune signaling are triggered by excess adiposity, which may be essential mediators of the metabolic dysfunction observed in obesity. However, not all obese individuals exhibit increased risk of inflammation and not all normal-weight individuals are metabolically healthy. A critical unanswered question is why and how the difference exists between metabolically healthy and unhealthy obese individuals regarding the obesity-associated inflammatory mediators. The answers to this question becomes even more important in the context of elderly, who present with diverse but more complex dimensions of body weight, patterns of body fat distribution, and obesity. This is why now, for example, there is great disagreement among the researchers whether to use the conventional BMI as an indicator of health outcomes or not as BMI is considered not sensitive enough to discriminate between lean body mass and body fat, particularly in elderly. Unfortunately, much of the previous work on the elderly focused on obesity assessed by BMI without considering the overall metabolic profile, which often yield controversial conclusions and misleading interpretations about obesity and its related comorbidities. Nevertheless, the association between obesity, morbidity, functional disability, and even mortality has been well established. It is noteworthy that the intensity of obesity-related inflammation may not be the same over age and unlike young, elderly may be victims of chronic inflammation beside acute inflammation, which results from persistent condition(s) in the elderly over the course of life-span including specific infections, CMV, for example. This state of chronic inflammation is often referred to as low-grade inflammation or even “inflammaging,” characterized by a variety of proinflammatory mediators with aging, namely, RANTES, MIP-1*α*, IL-8, MCP-1, IL-6, and TNF-*α* amongst others. As depicted in the paper, this resembles to some extent to the obesity-related inflammation. Individually, these mediators are involved in the development of many subclinical and clinically assessable events such as atherosclerosis, dementia, and diabetes in old age, which linked to a number of other comorbidities and the overall mortality. While the prevalence of most of current infections is decreasing, elderly individuals are more susceptible to infections, respond poorly to vaccination. The role of inflammation in immune erosion is to be considered seriously, especially when other chronic conditions such as obesity are present.
